# Lipid Bodies: Inflammatory Organelles Implicated in Host-*Trypanosoma cruzi* Interplay during Innate Immune Responses

**DOI:** 10.1155/2012/478601

**Published:** 2012-04-30

**Authors:** Heloisa D'Avila, Daniel A. M. Toledo, Rossana C. N. Melo

**Affiliations:** ^1^Laboratory of Cellular Biology, Department of Biology, Federal University of Juiz de Fora (UF JF), 36036-900 Juiz de Fora, MG, Brazil; ^2^Program of Cellular and Molecular Biology, Oswaldo Cruz Institute (IOC/FIOCRUZ), 21040-360 Rio de Janeiro, RJ, Brazil

## Abstract

The flagellated protozoa *Trypanosoma cruzi* is the causal agent of Chagas' disease, a significant public health issue and still a major cause of morbidity and mortality in Latin America. Acute Chagas' disease elicits a strong inflammatory response. In order to control the parasite multiplication, cells of the monocytic lineage are highly mobilized. Monocyte differentiation leads to the formation of phagocytosing macrophages, which are strongly activated and direct host defense. A distinguishing feature of Chagas' disease-triggered macrophages is the presence of increased numbers of distinct cytoplasmic organelles termed lipid bodies or lipid droplets. These organelles are actively formed in response to the parasite and are sites for synthesis and storage of inflammatory mediators. This review covers current knowledge on lipid bodies elicited by the acute Chagas' disease within inflammatory macrophages and discusses the role of these organelles in inflammation. The increased knowledge of lipid bodies in pathogenic mechanisms of infections may not only contribute to the understanding of pathogen-host interactions but may also identify new targets for intervention.

## 1. Introduction

The flagellated protozoa *Trypanosoma cruzi* is the causal agent of Chagas' disease (aka American trypanosomiasis) discovered at the beginning of the twentieth century by the Brazilian physician Carlos Chagas [1]. This disease remains a major problem with a great impact on public health in the Latin America. Chagas' disease affects nearly 8 million people and 28 million people are at risk of acquiring the disease in 15 endemic countries of Latin America [2]. Unfortunately, there is no vaccine available to prevent Chagas' disease [3].

*T. cruzi* is transmitted to humans primary through the feces of triatomine insects, at bite sites or in mucosa, through blood transfusion or orally through contaminated food. The parasite then invades the bloodstream and lymphatic system, and becomes established in the muscle and cardiac tissue, digestive system, and phagocytic cells [4]. *T. cruzi *may also be transmitted from mother to child across the placenta and through the birth canal, thus causing abortion, prematurity, and organic lesions in the fetus [4].

Chagas' disease is characterized by an acute phase with or without symptoms, and with entry point signs (inoculation chagoma or Romaña's sign), fever, adenomegaly, hepatosplenomegaly, evident parasitemia, and an indeterminate chronic phase (asymptomatic, with normal results from electrocardiogram and X-ray of the heart, esophagus, and colon) or with a cardiac, digestive, or cardiac-digestive form [4]. The factors that determine the distinct clinical outcomes, leading to a mild or to a severe form of the disease, are not completely understood. In fact, the most intriguing challenge to understanding the pathophysiology of Chagas disease still lies in the complex host-parasite interrelationship (reviewed in [5]).

The parasite has an obligate intracellular, proliferative, nonflagellate form, called amastigote. After many division cycles, the amastigote forms convert into a flagellate form, the infective trypomastigote. Due to the high number of parasites into the host cell cytoplasm, the cell membrane disrupts and the infection spreads, affecting different organs (reviewed in [4, 6]).

**Figure 1:**
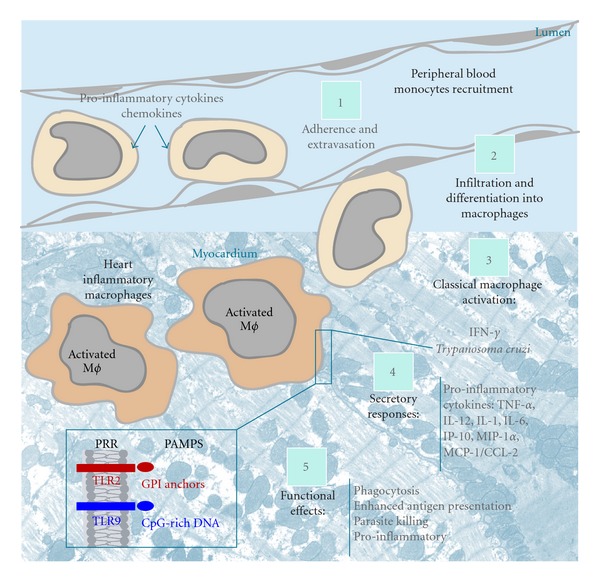
Activation of heart inflammatory macrophages during the acute infection with *Trypanosoma cruzi*. In response to acute infections, a cascade of events recruits cells derived from monocytic lineage from the peripheral blood into heart. This cascade culminates in a strong activation of macrophages. Classical activation of macrophages involves the key cytokine interferon gamma (IFN-*γ*) and *T. cruzi* components (GPI anchors and CpG-rich DNA). These parasite products are recognized at the macrophage surface by Toll-like receptors (TLRs)-2 and 9, respectively. These receptors are a class of pattern recognition receptors (PRRs), which initiate an immune response and directly activates macrophages. Additionally, TLR-2 can heterodimerizate either TLR1 or TLR6 to recognize *T. cruzi *GPI anchors [33]. TNF-*α* is one of the most efficient activators of macrophages to a trypanocidal function. PAMPS, pathogen-associated molecular patterns; GPI, glycosyl phosphatidy linositol; TNF-*α*, tumor necrosis factor-alpha; MIP-1*α*, macrophage inflammatory protein *α*; MCP-1/CCL-2, monocyte chemoattractant protein-1; IP-10, inducible protein 10.

**Figure 2:**
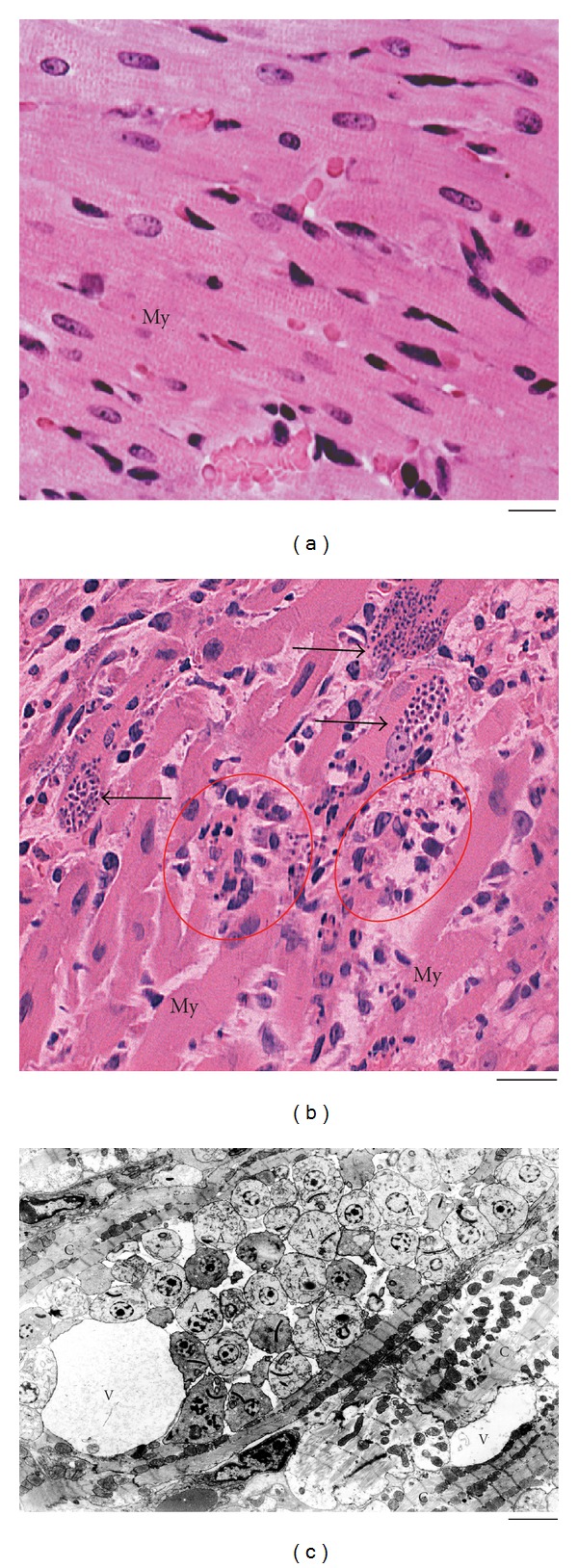
Morphological aspects of the heart from noninfected and infected rats at 12 days of infection with *Trypanosoma cruzi*. (a) Histological view of the myocardium from an uninfected rat. In (b), nests of amastigotes (arrows), the intracellular form of the parasite, and inflammatory processes (circles) characterized by predominance of mononuclear cells are observed in the myocardium (My). Semi-serial 5 *μ*m-thick sections of the heart were cut, stained by haematoxylin and eosin and examined for qualitative evaluation of the inflammatory and degenerative processes and quantification of parasitism [8, 34]. In (c), an electron micrograph of a parasitized cardiomyocyte (c) showing vacuoles (V) and many amastigotes (A). Data are representative of three independent experiments. Four to six rats per group. Panel (a) was reprinted from [9] with permission. Scale bar, 15 *μ*m (a, b); 1 *μ*m (c).

Acute Chagas' disease elicits a strong inflammatory response. In order to control the parasite multiplication, cells of the monocytic lineage are highly mobilized (Figure 1). There is an intense migration and extravasation of monocytes from the bloodstream into target organs, mainly the heart (Figures 1 and 2). Monocyte differentiation leads to the formation of phagocytosing macrophages, strongly activated and involved in inhibiting parasite replication in the myocardium and other tissues [7, 8] (Figure 1). The ability of activated macrophages to process and present antigens, produce cytokines, and provide costimulatory signals demonstrates their pivotal role in initiating immune responses. The importance of these cells to the host defense has been pointed out by us and other groups during the *in vivo* acute *T. cruzi* infection in both humans and experimental models [7–10].

 Because macrophages are key players in the initial resistance to the *T. cruzi* infection, a better understanding of their responses to the parasite is hence crucial for the development of appropriate therapeutic interventions and Chagas' disease control. A distinguishing feature of Chagas' disease-triggered macrophages is the presence of increased numbers of distinct cytoplasmic organelles termed lipid bodies (aka lipid droplets) [11]. Lipid bodies are lipid-rich organelles found in small numbers in most eukaryotic cells as roughly spherical organelles, comprised of an outer monolayer of phospholipids, a core containing neutral lipids, and variable protein composition. In contrast to other organelles, lipid bodies lack, therefore, a delimiting unit membrane structure (reviewed in [12]). Analysis of the fatty acid composition of the phospholipids revealed that they are structurally distinct from the phospholipids of the rough endoplasmic reticulum (ER) and from cholesterol/sphingolipid-rich microdomains. Unique features of lipid bodies include the abundance of unsaturated fatty acids in lyso-phosphatidylcholine and the relative abundance of phosphatidylcholine with 2 mono-unsaturated acyl chains [13]. The hydrophobic core of lipid bodies is occupied by triacylglycerols, diacylglycerols, retinyl esters, free cholesterol, and cholesterol esters in various ratios depending on the cell type [14–16].

 Leukocyte lipid bodies contain several functionally diverse types of proteins, including structural proteins, metabolic enzymes, and kinases. Lipid body-specific structural proteins, the PAT family of proteins—Perilipin, adipose-differentiation-related protein (ADRP) [17] and tail-interacting protein of 47 kDa (TIP47) [18]—are found at the circumferential rim of lipid bodies. Moreover, a number of small GTPases of the Rab family, considered critical regulators of vesicular traffic and organelle interaction, and a variety of other proteins are described in lipid bodies [17, 19, 20].

In the past, lipid bodies were largely associated with lipid storage, but it is now recognized that lipid bodies are dynamic and functionally active organelles linked to diverse biological functions, such as lipid metabolism, cell signaling, and membrane trafficking (reviewed in [12, 21]). Lipid body has also been associated to immunoregulatory function in a number of human inflammatory diseases including inflammatory arthritis [22], acute respiratory distress syndrome [23], hypereosinophilia syndrome [24], and mycobacterial infection [25, 26], in addition to be related to neoplasic and emerging metabolic diseases, such as atherosclerosis, diabetes, and obesity (reviewed in [27, 28]).

Since the first report on lipid body formation in response to the *in vivo* acute *T. cruzi* infection by our group in 2003 [11], we have been investigating these organelles as key players in the host-parasite interaction and markers of macrophage activation during infectious diseases [6, 29–31]. This review covers current knowledge on lipid bodies triggered by the acute Chagas' disease within inflammatory macrophages and discusses the role of these organelles in inflammation. The increased knowledge of lipid bodies in pathogenic mechanisms of infections may not only contribute to the understanding of pathogen-host interactions but may also identify new targets for intervention [12, 29, 31].

## 2. *Trypanosoma cruzi* Induction of Lipid Body Formation

 Although accumulation of lipid bodies has been documented for nearly 30 years in leukocytes and other cells in different inflammatory diseases [22–24, 32], the observation of lipid body formation in response to an *in vivo* infectious disease dates to 2003 [11]. By investigating inflammatory macrophages from rats infected with a virulent strain of *T. cruzi* (Y strain), it was observed a significant increase of the lipid body numbers in peritoneal macrophages at day 6 and 12 of the infection. While control peritoneal macrophages presented ~2.19 ± 0.4 (mean ± SEM) lipid bodies/macrophage, peritoneal macrophages from infected animals showed ~18.09 ± 1.4 at day 12 of infection [11]. 

**Figure 3:**
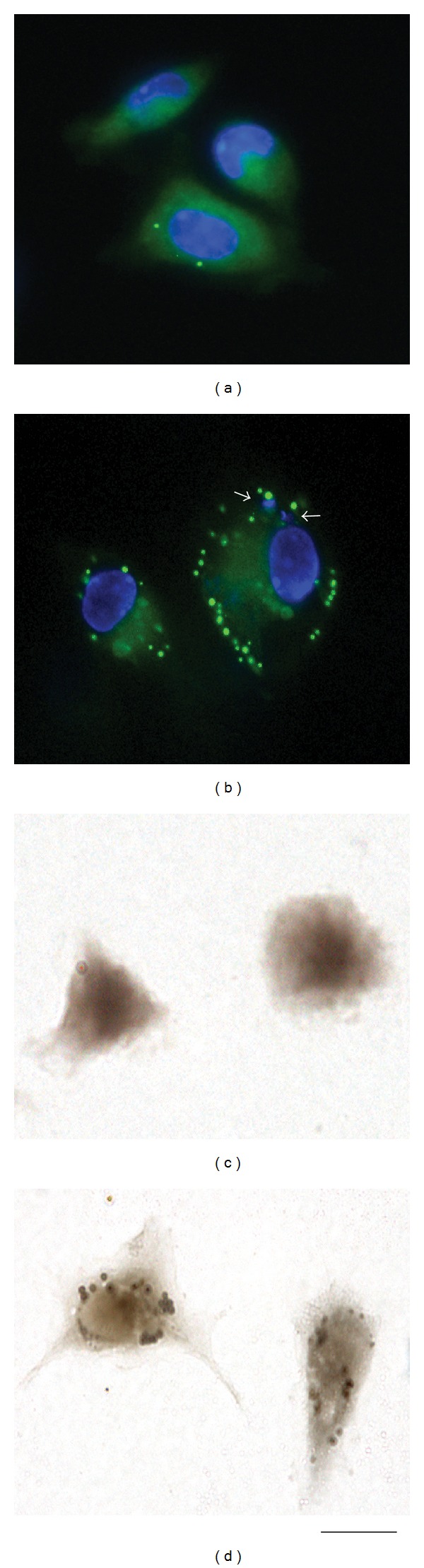
*Trypanosoma cruzi* infection induces macrophage lipid body formation. Peritoneal macrophages isolated from mice were cultured or not with *T. cruzi* and the formation of lipid bodies analyzed after 24 h by staining with BODIPY 493/503 (a and b), a fluorescent lipid probe for highly hydrophobic environments [35] or osmium tetroxide (c and d). While uninfected cells (a, c) have small number of lipid bodies, infected cells (b and d) show increased number of these organelles. Lipid bodies are seen as green (a and b) or brownish (c and d), round organelles. Nuclei of macrophages and internalized parasites (arrows) were stained with DAPI (4′,6-diamidino-2-phenylindole; blue). Scale bar, 10 *μ*m.

*In vitro*, lipid body accumulation has been observed within peritoneal macrophages isolated from mice and cultured with *T. cruzi* for 24 h [35] (Figures 3(a)–3(d)). At this time of infection, both the cells containing internalized parasites as well nonparasitized cells show increased number of lipid bodies compared to control, noninfected cells, suggesting a bystander amplification of the response (Figure 3(b)). Interestingly, parasitized cells show significant higher number of lipid bodies (threefold) compared to nonparasitized cells, indicating that the uptake of the parasite directly induces formation of lipid bodies [35] (Figure 3(b)). D'Avila and colleagues have also demonstrated that lipid body formation in macrophages in response to the *T. cruzi* infection occurs through a Toll-like receptor-2- (TLR2-) dependent mechanism, demonstrating a mechanism involving surface receptors in this event [35].

**Figure 4:**
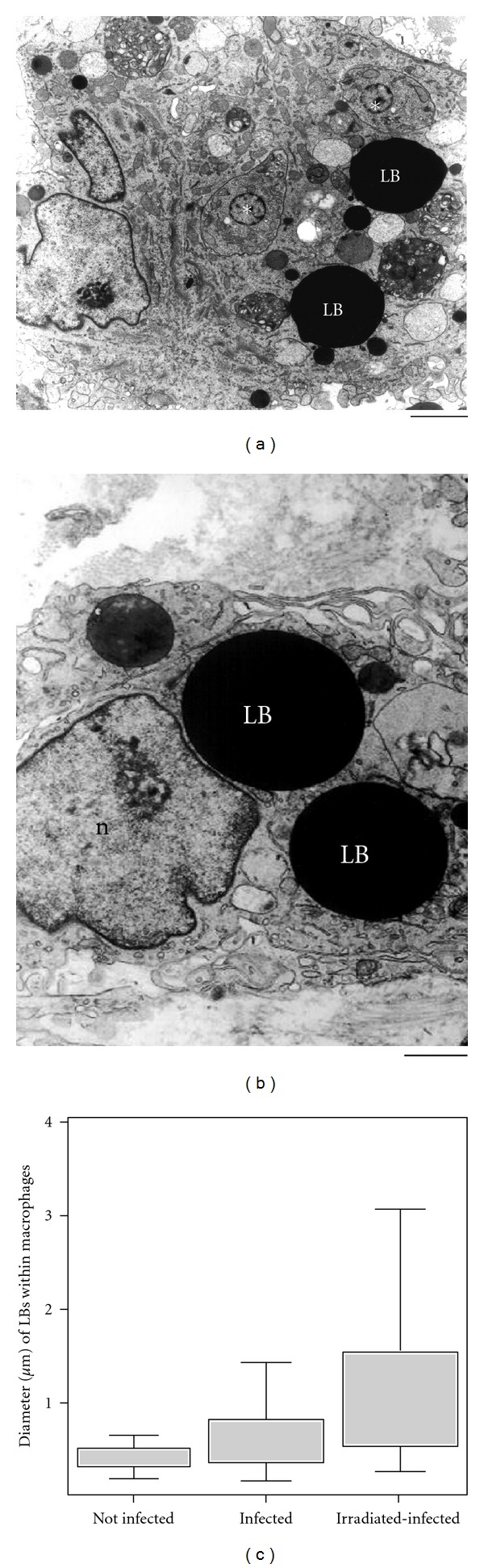
Lipid bodies (LB) within heart inflammatory macrophages increase in size in response to acute *Trypanosoma cruzi *infection and parasite load. (a) Strongly electron dense LBs from an infected animal are observed in the cytoplasm n conjunction with free amastigote forms of the parasite (asterisks). In (b), a giant LB is seen close to the nucleus in an irradiated-infected rat. (c) LB diameter variation in different groups. A significant increase of LB occurred in infected alone compared to uninfected and in irradiated-infected compared to infected alone groups (*P* < 0.05). Before infection, rats were irradiated or not and heart samples processed for transmission electron microscopy at day 12 of infection. Scale bar, 1.0 *μ*m (a); 600 nm (b). Reprinted from [29] with permission.

As noted, the accumulation of newly recruited macrophages in the heart is one of the main findings of the acute *T. cruzi* infection and it is correlated with the intense myocardium parasitism which occurs during the early infection in both experimental models and humans (reviewed in [6]) (Figure 2). At day 12 of *T. cruzi* infection in rats, is observed the most intense inflammatory process and parasitism in the heart compared to other points during the acute phase [7]. Ultrastructural analysis of this organ showed numerous infiltrating macrophages with lipid bodies prominently increased in number and size [11] (Figure 4(a)). Inflammatory heart macrophages, evaluated by quantitative electron microscopy, exhibited a mean of 8.3 lipid bodies/cell section (range of 1–25) at the same time of infection whereas control noninflammatory macrophages showed a mean of 2.6 lipid bodies/cell section (range of 0–3) [11].

One intriguing aspect of lipid bodies is their osmiophilia, which is dependent on the cell type and can change during elicited inflammatory responses (reviewed in [12]). In inflammatory macrophages, the lipid body density can consistently change during pathogen infections, as revealed by ultrastructural studies [29, 31]. Based on osmiophilia, lipid bodies were identified and quantitated as light-dense, electron-dense, and strongly electron-dense organelles within inflammatory macrophages from different origins, mainly from the heart [29]. *T. cruzi* infection induces a significant increase in the numbers of light-dense lipid bodies compared to noninfected controls which show lipid bodies preferentially as electron- dense organelles (Figure 4(a)). Of note, lipid bodies change consistently their osmiophilia in macrophages stimulated *in vivo* with higher parasite load in irradiated-infected rats. These animals were exposed to a single, high dose of gamma irradiation 1 day before infection, which depletes the humoral and cellular immune responses except for the phagocytic activity of macrophages. Inflammatory macrophages from irradiated-infected animals show an increase in the numbers of both light-dense and strongly electron-dense lipid bodies compared to infection alone [29] (Figures 4(b) and 4(c)). Lipid body morphological changes, including alterations in osmiophilia, may reflect differences in lipid composition, stages of formation of new lipid bodies, mobilization, and/or neutral lipids/phospholipids ratio within lipid bodies. In addition, these morphological changes highlight lipid bodies as dynamic organelles, able to consistently change their structure in concert with cell activation [29]. In fact, lipid bodies in activated macrophages can be imaged as heterogeneous organelles, with lucent areas, granular, and/or membranous internal structures [29]. Of interest, by proteomic and ultrastructural studies, we have defined lipid bodies as organelles with internal endoplasmic reticulum- (ER-) like membranes and ER luminal proteins, suggesting a model by which enveloped ER-membranes and domains form lipid bodies [19].

Another morphological feature of lipid bodies is their considerable size variation. For example, in scoring the diameters of lipid bodies within macrophages from rats experimentally infected with *T. cruzi*, 74% of lipid bodies had size <0.5 *μ*m in noninfected whereas 54% of lipid bodies from infected animals were >0.5 *μ*m, reaching up to 3 *μ*m (Figure 4(c)). Increase of the parasite burden induced by gamma irradiation triggered significant formation of large lipid bodies within inflammatory macrophages, with diameters around 4 *μ*m [29]. These findings reveal that not only the number but also the osmiophilia and size of lipid bodies represent structural indicatives of the participation of these organelles in innate immune responses [29].

Accumulation of lipid bodies within macrophages has also been documented during other infectious diseases, for example, during the progression of tuberculosis caused by *Mycobacterium tuberculosis* in both humans and experimental settings [25, 36] and in the course of leprosy, caused by *Mycobacterium leprae* [37]. In experimental studies with *Mycobacterium bovis *bacillus Calmette-Guérin (BCG), it was found that this pathogen is capable of inducing a dose- and time-dependent increase on LB formation within pleural and peritoneal macrophages [31, 38].

In addition to inducing lipid body formation [11], the infection with *T. cruzi* elicits a close interaction of lipid bodies with phagosomes within macrophages and even an apparent translocation of lipid bodies into these vacuoles, suggesting a discharge of the lipid body content [29]. However, the meaning of this interaction is still unknown. The lipid body-phagosome association as well as the internalization of lipid bodies by phagosomes during the acute *T. cruzi* infection may represent different stages of phagosome maturation leading to killing of the parasite or favor the parasite survival [29].

The lipid body-phagosome association has also been observed in cells infected with other pathogens such as *M. bovis* BCG [31], *M. tuberculosis* [39], and *Chlamydia trachomatis* [40]. It remains to be established if it represents a strategy for pathogen replication or if has implications for pathogen outcome. 

## 3. Lipid Body and Inflammation

As noted, accumulation of lipid bodies has been observed in the cytoplasm of activated cells associated with varied inflammatory and infectious conditions, both in clinical and experimental situations [23, 26, 31, 35, 36, 41, 42].

Lipid bodies are recognized sites for localization of arachidonic acid, the precursor for the synthesis of inflammatory mediators (eicosanoids) and eicosanoid-forming enzymes such as cyclooxygenases (COX) and lipoxygenases (LO) (reviewed in [12, 43]). The production of eicosanoids has been demonstrated during *T. cruzi* infection and both leukotrienes and prostaglandins seem to play a role in the pathogenesis of Chagas' disease. Macrophages, the first line of defense, are important sources for prostaglandin E_2_ (PGE_2_) production during *T. cruzi* infection [44–46] and parasite-induced lipid body formation within macrophages is accompanied by enhanced COX-2 expression [35]. In fact, by using fluorescence microscopy, it was demonstrated that *T. cruzi-*infected macrophages were found to be positive COX-2. The immunostaining appeared punctuate throughout the cytoplasm, suggesting that COX-2 may be localized within lipid bodies, in addition to the conventional perinuclear membrane localization. Colocalization of COX-2 and adipose differentiation-related protein (ADRP), a recognized marker for lipid bodies [47, 48], confirmed the presence of this enzyme within lipid bodies [35].

**Figure 5:**
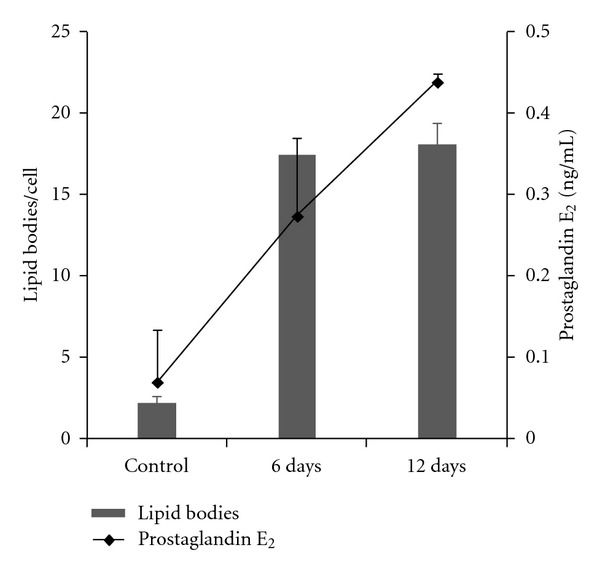
*Trypanosoma cruzi *infection induces concomitant lipid body formation and prostaglandin E_2_ (PGE_2_) synthesis. Associations between number of lipid bodies (bars) and prostaglandin E_2_ (PGE_2_) peritoneal levels (line) in rats at day 6 or 12 of infection with *T. cruzi *and in uninfected controls. At both days, the lipid body numbers were significantly increased (*P* < 0.05) in parallel to an accentuated increase of PGE_2_ synthesis. Data are expressed as means ± SEM. Reprinted from [11] with permission.

The increased formation of lipid bodies within inflammatory macrophages is accompanied by significant production of the PGE_2_. The highest numbers of lipid bodies induced by the *T. cruzi* infection in inflammatory macrophages occurred in parallel to the highest production of PGE_2_ [11] (Figure 5). This increase was documented at days 6 (fourfold) and 12 (sixfold) after infection in rats [11] (Figure 5). In murine model, the increased PGE_2_ production derived from lipid bodies was rapidly observed at 24 h of infection (fourfold), through a TLR2-dependent manner [35].

Prostaglandins down modulate a number of macrophage functions. Prostaglandins lead to reduced proinflammatory cytokine secretion, decreased antigen presentation, and diminished production of free radicals in these cells [49–52]. Studies have been demonstrating that prostaglandins are potent inhibitors of tumor necrosis factor-alpha (TNF-**α*) *synthesis and act as deactivators of macrophage trypanocidal function [46, 53]. Moreover, high levels of PGE_2_ favor *T. cruzi* replication and the treatment of the infected mice with nonsteroidal anti-inflammatory drugs (NSAIDs), inhibitors of COX-2 enzyme, significantly reduces parasite replication [35, 44, 54]. Interestingly, addition of exogenous PGE_2_ is also able to increase replication of the parasite *Leishmania amazonensis* in macrophages, indicating that PGE_2_ increases intracellular load of this pathogen in susceptible mice [55].

In contrast to inflammatory macrophages, peripheral blood monocytes from *T. cruzi*-infected animals show low number of lipid bodies [11]. The maturation of peripheral blood monocytes to tissue macrophages followed by activation of these cells is likely involved in lipid body formation and eicosanoid release during Chagas'disease [11].

Eicosanoids may also be directly observed within lipid bodies. By using a newly developed strategy, the Eicosacell methodology [56], which directly detects the eicosanoid synthesis *in situ*, intracellular sites of newly formed PGE_2_ colocalized with ADRP-labeled lipid bodies, confirming that lipid bodies are sites of compartmentalization of PGE_2_ synthesis during *T. cruzi* infection [35].

During the *T. cruzi* infection, the role of prostaglandins in the outcome of the parasite is still a matter of debate. It has been shown that the widely used nonsteroidal anti-inflammatory drugs (NSAIDs), aspirin (an inhibitor of both constitutive COX-1 and inducible COX-2) [57] and indomethacin (a preferential inhibitor of COX-1) [57], inhibit PGE_2_ synthesis and are able to control parasitaemia in susceptible mice [44]. Likewise, aspirin and NS-398 (COX-2 inhibitor) [58]) were able to modulate lipid body formation and consequently to inhibit the PGE_2_ production and parasite growth in macrophages [35]. These data support the concept that *T. cruzi* induces and exploits host-derived lipid bodies to extend and maintain its own survival. Moreover, pharmacologic intervention of lipid body biogenesis inhibits *T. cruzi* survival and replication in macrophages, and therefore these organelles may act as potentially targets for therapy during the acute phase of Chagas' disease [35].

## 4. Lipid Body and Apoptosis during *T. cruzi *Infection

Apoptosis of host cells, mostly lymphocytes and cardiomyocytes, has been identified during the *T. cruzi* infection in both humans and experimental models and seem to play an important immune regulatory role in this and other parasitic infections [44, 59, 60].

**Figure 6:**
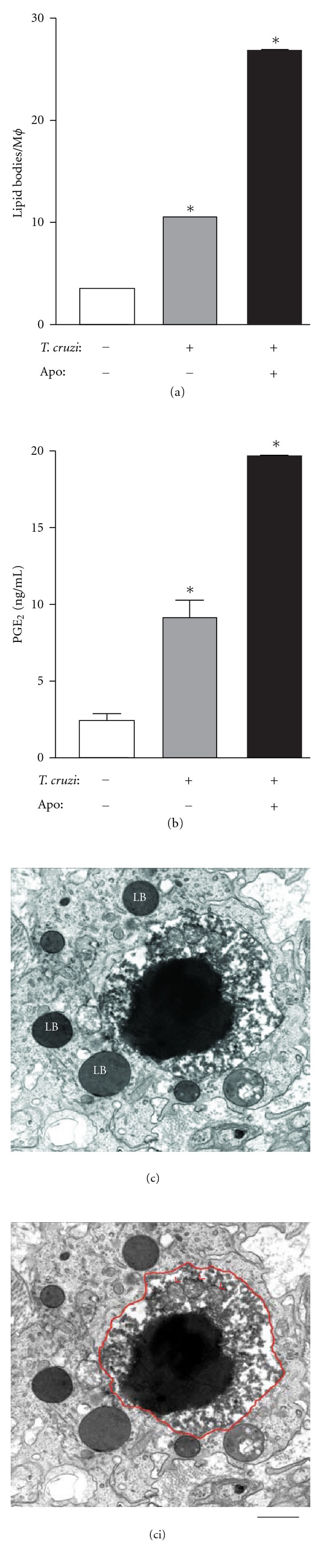
Uptake of apoptotic cells by macrophages induces lipid body formation and PGE_2_ synthesis during *Trypanosoma cruzi* infection. LB formation (a) and prostaglandin E_2 _(PGE_2_) synthesis (b) by mice macrophages infected *in vitro* with *T. cruzi* alone or co-cultured with apoptotic cells for 24 hours. Each bar represents the mean ± standard error of the mean (SEM) from 50 consecutively counted macrophages from at least 4 independent pools of 3 animals each. Statistically significant (*P* # 0.05) differences between control and infected or stimulated groups are indicated by asterisks; M*ϕ*, macrophages. In (c), a large typical apoptotic body (outlined in red in (ci)) showing very condensed, electron-dense nuclear chromatin and degenerating mitochondria (red arrowheads) is seen within a heart macrophage of a rat with 12 days of infection. Note that the apoptotic body is surrounded by several lipid bodies with distinct electron densities. Rats were infected with the Y strain of *T. cruzi* and the myocardium processed for transmission electron microscopy at day 12 of the acute infection. Reprinted from [35] with permission. Scale bar, 1 *μ*m.

Uptake of apoptotic bodies, a process termed efferocytosis, is able to impact on host inflammatory mediator production and susceptibility to infection [44, 60, 61]. It was recently demonstrated that efferocytosis may affect lipid body formation and PGE_2_ synthesis during the *T. cruzi* infection [35] (Figure 6). These authors showed that the uptake of apoptotic cells, but not living or necrotic cells by cultured macrophages, triggers lipid body formation in the absence of infection. However, when infected macrophages are exposed to apoptotic cells, the efferocytosis process amplifies the effects of the parasite on lipid body formation leading to a higher lipid body accumulation compared to noninfected cells exposed to apoptotic bodies (Figure 6). Consistent with the lipid body function in inflammation, as discussed in Section 3, the uptake of apoptotic cells by both infected and noninfected macrophages induces increased lipid body-derived PGE_2_ synthesis. Interestingly, 24 h after the uptake of apoptotic cells by noninfected macrophages, newly formed lipid bodies show *in situ* both COX-2 and PGE_2_, similarly to infected cells [35].

Freire de Lima and colleagues [44] demonstrated that the recognition of apoptotic cells by the *α*_v_ *β*_3_ integrin (vitronectin receptor) is decisive for apoptotic-cell cytoadherence and the induction of both PGE_2_ and transforming growth factor beta (TGF-*β*) release during *T. cruzi* infections. The involvement of *α*_v_ *β*_3_ integrin on lipid body formation and parasite replication induced by apoptotic cell uptake was also evaluated utilizing flavoridin, a desintegrin that blocks binding of integrins *α*_v_ *β*_3_ [62]. Flavoridin blocked lipid body formation and COX-2 expression, induced by incubation of macrophages with apoptotic cells, indicating that the engagement and activation of *α*_v_ *β*_3_ is sufficient to trigger lipid body biogenesis, COX-2 expression, and enhanced PGE_2_ synthesis in macrophages [35]. 

Distinct works have been demonstrating that TGF-*β* is consistently produced during the *T. cruzi* infection [35, 63, 64]. Moreover, as noted, the interaction of macrophages with apoptotic cells leads macrophages to produce TGF-*β* and also renders phagocytic cells more permissive to *T. cruzi* infection [35, 63–65]. A recent report showed that TGF-*β* induces lipid body formation, affecting in turn the PGE_2_ release, and making phagocytic cells more permissive to *T. cruzi* infection [35]. In fact, in conjunction with increased lipid body formation and PGE_2_ production, infected macrophages presented exacerbated parasite replication when cocultured with apoptotic cells for 24 hours [35]. The capacity of other cytokines to modulate lipid body formation in macrophages during the *T. cruzi *infection remains to be defined. 

As mentioned in Section 3, aspirin and NS-398 inhibit cyclooxygenase production. Likewise, these drugs are able to inhibit lipid body formation in infected macrophages in the presence or absence of apoptotic cells, suppresses apoptotic cell effects on lipid body-derived PGE_2_ production, and reverses the effects of apoptotic cells on parasite replication [35]. Accordingly, the fatty acid synthase inhibitor C75 significantly inhibited lipid body formation induced by *T. cruzi*, with or without the presence of apoptotic cells. Strikingly, it was demonstrated that the treatment with C75, in parallel to lipid body inhibition, reversed the parasite replication induced by apoptotic cells [35]. In summary, macrophage lipid bodies formed during *T. cruzi* infection in association with apoptotic cell stimulation directly impact the capacity of macrophages to produce increased amounts of PGE_2_, which may have impact on the ability of the host to control the infection.

## 5. Final Remarks

In recent years, the association of lipid bodies, morphologically distinct organelles, with many potential roles in cells, in both health and disease, has brought special attention to them. Lipid body accumulation has been documented in varied inflammatory situations, in both experimental and human conditions [12, 25, 66].

Research over the last decade has identified an important formation of lipid bodies within inflammatory macrophages in response to the *T. cruzi*-host interaction. Newly formed lipid bodies during Chagas' disease and other inflammatory diseases are notable for their capacity to synthesize inflammatory mediators, such as PGE_2_ and for expressing enzymes linked to this synthesis, such as COX-2 [12, 21]. Lipid bodies elicited by *T. cruzi* and other pathogens are now recognized not only as inflammatory organelles and structural markers of pathogen-induced cell activation, but also as organelles able to modulate host cell processes [11, 29, 35]. For example, a recent work supports the concept that *T. cruzi* induces and exploits host-derived lipid bodies to extend and maintain its own survival [35]. However, much remains to be learned. More work will be needed to understand the influence of lipid bodies on the host cell physiology and if these organelles have a major role in the destruction or intracellular survival of the parasite. The increased knowledge of lipid bodies in pathogenic mechanisms of infections may not only contribute to the understanding of pathogen-host interactions but may also identify new targets for intervention.
